# Voltage-Gated Potassium Channel Kv1.3 as a Target in Therapy of Cancer

**DOI:** 10.3389/fonc.2019.00933

**Published:** 2019-09-24

**Authors:** Andrzej Teisseyre, Anna Palko-Labuz, Kamila Sroda-Pomianek, Krystyna Michalak

**Affiliations:** Department of Biophysics, Wroclaw Medical University, Wrocław, Poland

**Keywords:** Kv1.3 channel, cancer, proliferation, apoptosis, Kv1.3 channel inhibitors

## Abstract

Voltage-gated potassium channel Kv1.3 is an integral membrane protein, which is selectively permeable for potassium ions and is activated upon a change of membrane potential. Channel activation enables transportation of potassium ions down their electrochemical gradient. Kv1.3 channel is expressed in many cell types, both normal and cancer. Activity of the channel plays an important role in cell proliferation and apoptosis. Inhibition of Kv1.3 channel may be beneficial in therapy of several diseases including some cancer disorders. This review focuses on Kv1.3 channel as a new potentially attractive molecular target in cancer therapy. In the first part, changes in the channel expression in selected cancer disorders are described. Then, the role of the channel activity in cancer cell proliferation and apoptosis is presented. Finally, it is shown that some low molecular weight organic inhibitors of the channel including selected biologically active plant-derived polycyclic compounds may selectively induce apoptosis of Kv1.3-expressing cancer cells while sparing normal cells and healthy organs. These compounds may be promising candidates for putative application in therapy of some cancer disorders, such as melanoma, pancreatic ductal adenocarcinoma (PDAC), or B-type chronic lymphocytic leukemia (B-CLL).

## Introduction

Voltage-gated potassium channels (Kv) are integral membrane proteins, which are selectively permeable for potassium ions and are activated upon a change of cell membrane voltage. Activation of these channels provides transportation of potassium ions across cell membrane down their electrochemical gradient. Kv channel of the Kv1.3 type was firstly discovered in the plasma membrane of human T lymphocytes (1, 2). Activation of Kv1.3 channel in the plasma membrane provides an efflux of potassium ions out of the cell and stabilization of the resting membrane potential (2, 3). Kv1.3 channel is expressed in human T and B lymphocytes, macrophages, fibroblasts, platelets, macrophages, osteoclasts, microglia, oligodendrocytes, brain (e.g., olfactory bulb, hippocampus, and cerebral cortex), lung, islets, thymus, spleen, lymph nodes, and testis (3). Kv1.3 channel is also expressed in the inner mitochondrial membrane (mito Kv1.3) of normal human T lymphocytes and cancer cells, such as human leukemic T cell line Jurkat, prostate cancer PC-3 cells and breast cancer MCF-7 cells (4, 5). Recently published data demonstrate that Kv1.3 channel is also expressed in the nuclei of cancer cells, such as Jurkat T cells, breast cancer MCF-7, lung cancer A549 and gastric cancer SNU-484 cells as well as in human brain tissues (6). Moreover, Kv1.3 channel was also discovered in the cis-Golgi apparatus membrane in rat cancer astrocytoma C6 cells as well as in non-cancerous CTX TNA2 astrocyte cell line and in rat primary astrocytes (7). Activity of Kv1.3 channel plays an important role in cell proliferation and apoptosis (3, 5, 8–14). The channel activity is inhibited by many chemically unrelated compounds: heavy-metal cations, small-molecule organic compounds and venom-isolated oligopeptides (3, 8–14). The most potent specific inhibitors inhibit the channel at subnanomolar concentrations (3, 8–14). Inhibition of Kv1.3 channel by specific inhibitors may be beneficial in therapy of T-lymphocyte-mediated autoimmune diseases (e.g., sclerosis multiplex, type I diabetes mellitus, rheumatoid arthritis, psoriasis), chronic renal failure, asthma, obesity, type II diabetes mellitus, cognitive disabilities, and some cancer disorders (8–15). This review paper is focused on Kv1.3 channel as a new, potentially attractive molecular target in cancer therapy. In the first section the changes in Kv1.3 channel expression in cancer cells are described. Then, a role of activity of Kv1.3 channel in cancer cell proliferation and apoptosis is reviewed. It is pointed out how inhibition of Kv1.3 channel may inhibit uncontrolled cancer cell proliferation and induce apoptosis of cancer cells. In the last section, inhibitors of Kv1.3 channel that may be promising candidates for a putative application in therapy of some cancer disorders are presented.

## Changes in Expression of Kv1.3 Channel in Cancer

Several studies have demonstrated an altered expression of Kv1.3 channel in case of some cancer disorders when compared to normal tissues (15–19). However, no general pattern of these changes is known at the present. The changes depend on the type and the stage of the disease. In cancer tissues these channels may be up-regulated or down-regulated (Table 1). An up-regulated expression of the channel was discovered in breast or colon cancer, in smooth muscle (leiomyosarcoma), skeletal muscle (alveolar rhabdomyosarcoma), lymph node cancer (15–19), and in mature neoplastic B cells in chronic lymphocytic leukemia (B-CLL) (20).

**Table 1:** Changes in the expression of Kv1.3 channels in selected cancer disorders.

**Type of a cancer disorder**	**Changes in the expression of Kv1.3 channels**	**References**
Chronic B lymphocytic leukemia (B-CLL)	Up-regulation in mature neoplastic B cells probably due to a haploinsufficiency of the KCNRG gene and to an up-regulation of B-RAF kinase.	(20)
Acute T—cell leukemia	Up-regulation in leukemic cell lines: Jurkat T, CEM, and MOLT-3 probably due to an up-regulation of B-RAF kinase.	(20–22)
Breast adenocarcinoma	Up-regulation in highly tumorigenic M13SV1R2-N1 cells compared to weakly tumorigenic M13SV1R2 cells and normal mammary epithelial cells M13SV1. Down-regulation in patients correlated with the disease's grade due to the methylation of the gene's promoter region.	(23, 24)
Prostate cancer	Channels' expression is significantly higher in weakly metastatic LNCaP and AT-2 cell lines than in strongly metastatic PC3 and Mat-LyLu cells. Down-regulation in patients correlated with the disease's grade and stage.	(25–27)
Pancreas adenocarcinoma	Up-regulation in pancreas ductal adenocarcinoma (PDAC) cell lines: As PC-1, Capan-1, Panc-1, Mia PaCa 2, Bx PC-3, and Colo357. Down-regulation in patients correlated with the metastasis due to the methylation of the gene's promoter region.	(28, 29)
Colorectal cancer	Down-regulation in patients due to the methylation of the gene's promoter region. Significant reduction of the 5-year overall survival rate (OS) due to the gene's methylation.	(30)

In case of B-CLL it was shown that these cancer cells express significantly more channels than normal human B lymphocytes (20). This up-regulation may be due to a haploinsufficiency of the potassium channel regulating gene (KCNRG), which causes suppression of potassium channel expression and activity (20). Moreover, it was shown that inhibition of B-RAF kinase by a potent anti-proliferative and pro-apoptotic compound PLX-4720 significantly reduced the expression of Kv1.3 channel in cancer B-CLL, but not in normal B cells (20). A similar reduction of Kv1.3 channel expression upon application of PLX-4270 was also observed in case of human leukemic Jurkat T cells (20). These results suggest that B-RAF activity is involved in up-regulation of Kv1.3 channel in leukemic B and T cells. This B-RAF-dependent increased expression of Kv1.3 channel in cancer cells may be related to a pro-proliferative effect on these cells (20). A significantly up-regulated expression of Kv1.3 channel that was positively correlated with tumor aggressiveness was recently observed in case of a leiomyosarcoma and alveolar rhabdomyosarcoma (19, 31).

In case of breast cancer, a significantly up-regulated expression of Kv1.3 channel mRNA in cancer cells was already observed in the I-st stage of the disease (23). Moreover, it was observed that tumorigenic mammary epithelial M13SV1R2 and M13SV1R2-N1 cells expressed significantly more channel proteins than normal human mammary epithelial cells M13SV1 (23). Among cancer cells, the channel expression was significantly higher in highly tumorigenic M13SV1R2-N1 cells than in weakly tumorigenic M13SV1R2 cells (23). On the other hand, a markedly reduced expression of Kv1.3 channel at both mRNA and protein levels was detected in case of breast adenocarcinoma and an inverse correlation between the channel expression and grade of the disease was obtained (24).

A significantly reduced expression of the channel was also observed in cancer of kidney, bladder, pancreas, lung, brain (astrocytoma, oligodendroglioma, and glioblastoma), stomach and prostate (15–19, 24, 25, 28). In case of prostate cancer there was a significant inverse correlation between expression of the channel in the epithelium of human prostate tissue and grade (*p* = 0.003) and stage (*p* = 0.001) of the tumor. However, no correlation was observed between expression of Kv1.3 channel and race or age of the patients (25). Overall, immunostaining studies showed a strong (staining score 4 or 5) expression of Kv1.3 channel in the prostate epithelium in the whole control group, in almost all (17/18) patients with benign prostatic hyperplasia (BPH), but only in 52% (77/147) specimens with primary human prostate cancer (Pca) (25). Furthermore, the highest (5) staining score was observed in 60% (6/10) of controls, 67% (12/18) of BPH patients and only in 20% (29/147) of Pca specimens (25). Moreover, in prostate cancer, the channel expression is significantly higher in weakly metastatic LNCaP and AT-2 cell lines than in strongly metastatic PC3 and Mat-LyLu cell lines (26, 27). A significant reduction of the expression of Kv1.3 channel was also observed in case of pancreas adenocarcinoma, especially in patients with metastasis (28). It was shown that the reduction of channel expression in the case of breast and pancreas adenocarcinoma was a consequence of methylation of the promoter region of Kv1.3-encoding gene in cancer cells. This process was enhanced in patients with metastasis (24, 28). In case of breast adenocarcinoma, it was shown that Kv1.3 gene promoter was methylated only in 12.5% (2/16) of patients with the Grade I of the disease (SBR Histological Grade), but in 52.2% (12/23) and in 53.9% (7/13) of patients with the Grade II and III, respectively (24). There was a positive correlation between the gene methylation and the grade of the disease, but a negative correlation was obtained between the grade of disease and expression of Kv1.3 mRNA and channel protein (24). Moreover, Kv1.3 gene promoter was methylated in MCF-7 breast carcinoma cell line whereas the methylation was absent in a primary culture of normal breast cells (HMEpC) (24). The gene methylation was also detected in case of 76.9% (20/26) of patients with pancreas cancer metastasis, but only in 40% (2/5) of patients without metastasis (28). The survival distribution function showed that the gene methylation reduced the expected mean survival time for patients suffering from pancreas cancer from more than 2 years to about 1 year (28). Recently published data showed that the complete gene methylation was responsible for lack of expression of Kv1.3 channel in colorectal cancer cell lines: RKO, DLD-1, SW-620, HCT-116, and HT-29 (30). On the other hand, the channel was expressed in LoVo and SW-480 cells, where the gene methylation occurred only partially (30). Moreover, it was shown that application of the gene demethylation agent: 5-aza-2'-deoxycytidine (DAC) restored the channel expression in RKO, DLD-1, SW-620, HCT-116, and HT-29 cells (30). Clinical tests showed that the gene methylation occurred in case of 76.9% (112/147) of primary colorectal cancer, whereas no methylation was detected in normal colorectal mucosa (30). It was shown that Kv1.3 channel expression was reduced in 76.7% (23/30) of colorectal cancer patients; in almost all cases (22/23) it was due to the gene methylation (30). The gene methylation also reduced 5-year overall survival rate (OS) of colorectal cancer patients from 80 to 64.3% (30). Thus, both expression of Kv1.3 channel and Kv1.3 gene promoter methylation may serve as diagnostic and prognostic marker in case of breast, pancreas, and colorectal cancer. However, in case of some disorders, such as in brain tumors (astrocytoma, oligodendroglioma, and glioblastoma) a clear correlation between the channel expression and stage of the disease is still not established (19).

## Role of Kv1.3 Channel in Cancer Cell Proliferation

It is well-known that the activity of Kv1.3 channel is needed for proliferation of Kv1.3 channel-expressing normal and cancer cells. It was shown that activated human naive and central memory human T lymphocytes (T_CM_) up-regulate both Kv1.3 channel and calcium-activated potassium channel K(Ca)3.1, whereas effector memory T lymphocytes (T_EM_) predominantly up-regulate Kv1.3 channel (8–14). Thus, a selective inhibition of Kv1.3 channel can selectively suppress activated T_EM_ cells, whereas activated naive and T_CM_ cells can escape from the suppression due to the up-regulation of K(Ca)3.1 channel (8–14). This is the idea of a “selective immunosuppression,” which is a promising approach in in therapy of T-lymphocyte-mediated autoimmune diseases, such as, for example, sclerosis multiplex, type I diabetes mellitus, rheumatoid arthritis, or psoriasis (8–14). It is known that human leukemic Jurkat T cells express both Kv1.3 channel and apamin-sensitive calcium-activated potassium channel K(Ca)2.2 (21). Recently published results show that both Kv1.3 and K(Ca)3.1 channels are up-regulated in two leukemic cell lines: CEM and MOLT-3 cells (22). Moreover, as it was mentioned above, an over-expression of Kv1.3 channel, in relation to normal human B lymphocytes, occurs also in case of mature neoplastic B cells in chronic lymphocytic leukemia (B-CLL) (20).

**Figure 1:**
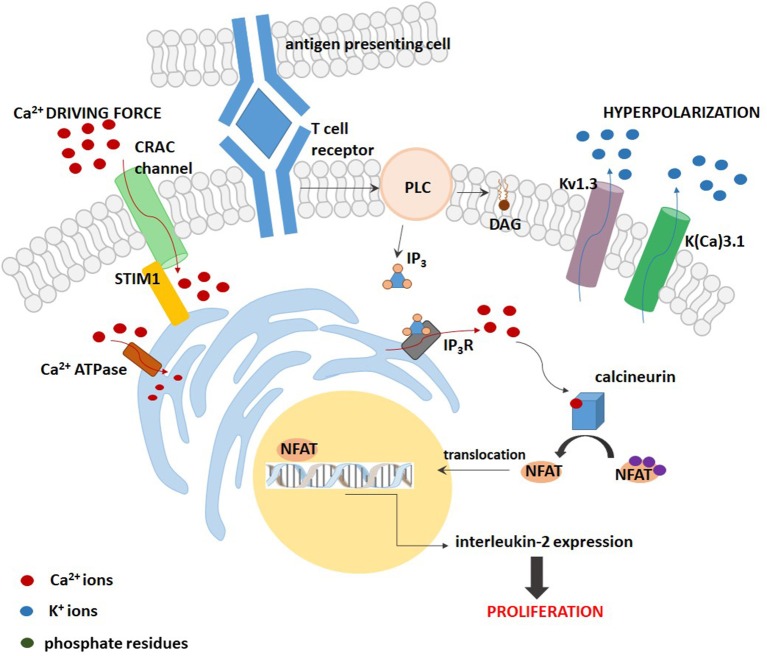
A scheme of the “membrane potential model” for the contribution of Kv1.3 and K(Ca) channels to proliferation of T lymphocytes.

Two different models can be applied to describe the role of Kv1.3 channel in regulation of cell proliferation (32). First of the models—the “membrane potential model”—was firstly described in T lymphocytes (Figure 1). In these cells, Kv1.3 channel is a part of the “immune synapse” between T Cell Receptor (TCR) and Antigen Presenting Cell (APC). Activation of TCR leads to an activation of the enzyme phospholipase C (PLC), which catalyzes the production of diacylglycerol (DAG) and inositol-1,4,5-triphosphate (IP_3_). The later compound binds to its receptor on the endoplasmic reticulum (ER), which is a calcium-selective receptor-gated channel, and thereby promotes release of calcium ions from the ER. The calcium release is followed by a sustained calcium influx due to an activation of voltage-independent calcium release-activated CRAC channel coupled to the ER by a STIM1 protein in the plasma membrane (32). This calcium ions influx is necessary for a subsequent activation of the protein phosphatase calcineurin which de-phosphorylates the Nuclear Factor of Activated T Cells (NFAT). The de-phosphorylated NFAT is translocated to the nucleus, where it binds to the promoter of interleukin-2 (IL-2) gene. This promotes synthesis and release of IL-2, which is a T-lymphocyte growth factor (32, 33). The production of IL-2 provides T lymphocyte proliferation even in the absence of antigen (32). According to the “membrane potential model,” an alternate opening of Kv1.3 and K(Ca)3.1 channels leads to an efflux of potassium ions out of the cell and thereby to a hyperpolarisation of the plasma membrane. Due to alternate channel activation the membrane potential is fluctuating around average value. This fluctuating hyperpolarisation provides a creation of a fluctuating electrochemical “driving force” for calcium influx through CRAC channels. Inhibition of Kv1.3 and K(Ca)3.1 channels causes a depolarisation of the plasma membrane and reduction of the “driving force” for calcium entry. This caused an inhibition of the whole calcineurin-NFAT signaling pathway and inhibition of IL-2 synthesis (33). Inhibition of IL-2 production arrests in turn cell cycle in the G_1_ phase and thereby inhibits cell proliferation (8–14, 32).

**Figure 2:**
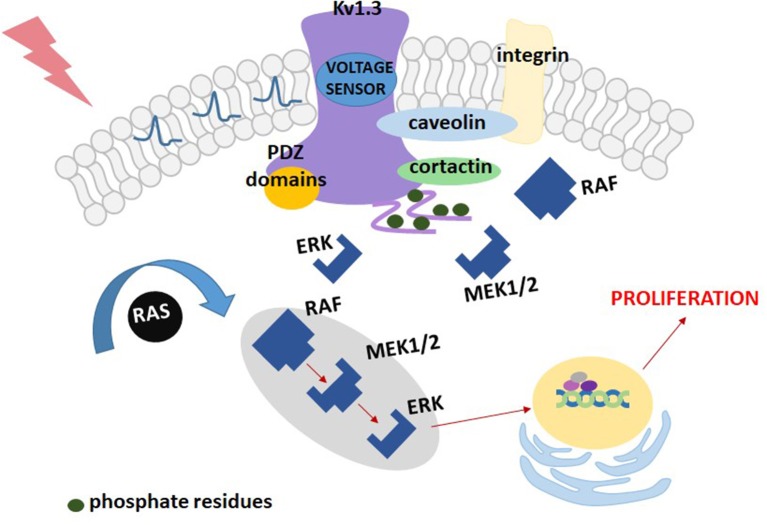
A scheme of the “voltage sensor model” for the contribution of Kv1.3 channel to proliferation of Kv1.3-transfected HEK293 cells.

The “membrane potential model” may be oversimplified and not adequate for cells other than lymphocytes. If the role of Kv1.3 channels in cell proliferation would only be associated with stabilization of the cell membrane potential, the same role could also be played by other types of voltage-gated potassium channels, such as for example, Kv1.5 channel, structurally related to Kv1.3 (32). The results obtained with HEK 293 cells demonstrated that transfection of these cells with plasmids containing Kv1.3 channel gene stimulated cell proliferation, whereas the same operation done with Kv1.5 channel gene inhibited the proliferation (34, 35). These results cannot be explained by the “membrane potential model,” since both types of channels have a similar influence on the cell membrane potential. Therefore, another model of the influence of Kv1.3 channel on cell proliferation known as the “voltage sensor model” was elaborated [(32, 34, 35), Figure 2]. According to this model, Kv1.3 channel works as membrane voltage sensor that is sensitive to changes in membrane voltage upon cell stimulation (32). Depolarisation of the cell membrane causes opening of the channels and this opening promotes phosphorylation of intracellular C-terminal tyrosine (Y) and serine (S) residues: Y447, S459, S473, S475, and Y477 (34, 35). The residues are phosphorylated by protein kinases being a part of the MEK-ERK signaling pathway (35). This phosphorylation is required for activation of the MEK-ERK signaling pathway, however, the mechanism of interactions between the phosphorylated channel and the signaling pathway remains to be elucidated.

Interestingly, according to this model, a key factor necessary to promote cell proliferation is not generation of the “driving force” for calcium influx, but the channel opening. The proliferation is inhibited only if inhibitor prevents the channel from opening (34, 35).

Taking into account these contradictory models one can conclude that the mechanism of participation of Kv1.3 channel in cell proliferation is probably complex and it may contain two components: setting of the cell membrane potential leading to generation of the “driving force” for calcium influx, and an activation of the MEK-ERK signaling pathway.

## Role of Kv1.3 Channel in Cancer Cell Apoptosis

Kv1.3 channel also participates in induction of apoptosis of normal and cancer cells that express this channel (5, 11–14).

**Figure 3:**
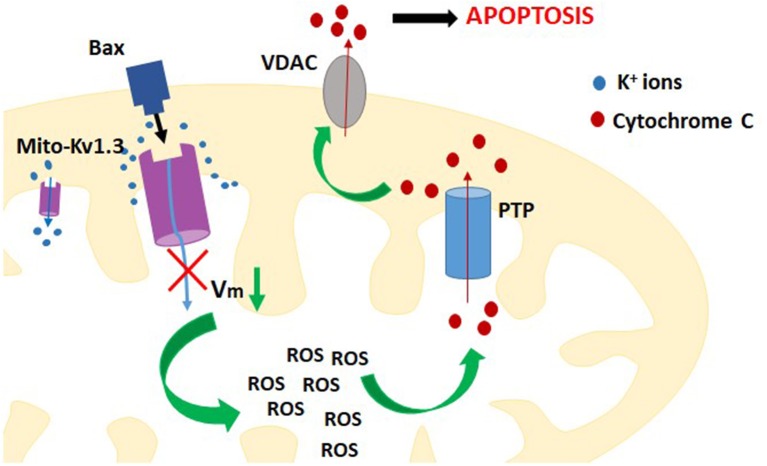
A scheme of the contribution of inhibition of mito-Kv1.3 channel to activation of intracellular (mitochondrial) pathway of apoptosis of Kv1.3 channel expressing cells.

Studies performed on Jurkat T cells showed that activity of Kv1.3 channel in the plasma membrane was up-regulated upon activation of death receptor pathway of apoptosis by Fas ligands (36). The up-regulation of the channel activity occurred via caspase 8-dependent pathway. It generated a sustained efflux of potassium ions out of the cell and cell shrinkage, which is a hallmark of apoptosis (36). It is also known that expression of mito Kv1.3 channel is required to induce the mitochondrial pathway of apoptosis by the pro-apoptotic protein Bax (37). It was shown that Bax, which is cumulated in the outer mitochondrial membrane upon apoptotic stimulation, directly inhibits mito Kv1.3 channel by a peptide toxin-like mechanism (37). The EC_50_ value for the inhibition by Bax is about 4 nM. This indicates that Bax is among most potent inhibitors of Kv1.3 channel. The inhibition of mito Kv1.3 channel is the first crucial step in the activation of mitochondrial pathway of apoptosis (Figure 3). Since the activation of mito Kv1.3 channel leads to a generation of an inward potassium current, inhibition of this channel would cause a hyperpolarisation of the inner mitochondrial membrane. Experiments performed on isolated mitochondria showed that inhibition of mito Kv1.3 channel by Bax caused a transient hyperpolarisation of the inner mitochondrial membrane and this hyperpolarisation was followed by a pronounced depolarisation (5, 37). It is known that hyperpolarisation of the inner mitochondrial membrane facilitates the production of reactive oxygen species (ROS) by mitochondria (38). Increased production of ROS activates the mitochondrial permeability transition pore (PTP) probably by oxidation of cysteine residues (5, 37). Activation of the PTP leads to a loss of the mitochondrial membrane potential. Moreover, increased ROS level triggers detachment of cytochrome c probably due to oxidation of membrane lipids. Detached cytochrome c is then released from the mitochondrial space through the PTPs in the inner mitochondrial membrane and voltage-dependent anion channels (VDAC) in the outer mitochondrial membrane (39). Release of cytochrome c was also observed upon Bax application in Kv1.3 channel- expressing cells (5, 37). Increased production of ROS, depolarization of the inner mitochondrial membrane, cytochrome c release—all these processes are hallmarks of activation of mitochondrial pathway of apoptosis.

It is well-known that cancer cells may develop resistance to apoptosis. This resistance can be achieved, for example, by down-regulation of Kv1.3 channel, including the mito Kv1.3, due to methylation of the promoter region of Kv1.3-encoding gene. Such a process was enhanced in patients with metastasis (see above). However, it was shown that expression of the channel in colorectal cancer cells was restored upon an application of the gene demethylation agent (30). Restoration of the channel expression may re-sensitize these cells to apoptotic stimuli. This was observed in case of Kv1.3-deficient murine cytotoxic T lymphocyte cell line CTLL-2, which is resistant to the mitochondria-mediated apoptosis (5, 37) Restoration of expression of Kv1.3 channel in these cells by Kv1.3 vector transfection re-sensitized these cells to apoptotic signals (5, 37). Alternatively, cancer cells may become resistant to apoptosis by a down-regulation of pro-apoptotic Bax proteins combined with an up-regulation of anti-apoptotic Bcl proteins. A mutational inactivation-mediated deficiency of pro-apoptotic proteins Bax and Bak, which is often observed in tumor cells, can protect them from mitochondria-mediated apoptosis induced by anticancer drugs such as: etoposide, bleomycin, or cisplatin (40). Nevertheless, the pro-apoptotic effect of Bax in isolated cancer cell mitochondria was mimicked upon application of a potent and selective Kv1.3 channel inhibitor—margatoxin (MgTX) (37). Unfortunately, MgTX cannot block mito Kv1.3 channel in intact cells, because it is membrane-impermeant. Therefore, it is of importance to discover small-molecule, selective and membrane-permeant inhibitors, which can inhibit mito Kv1.3 channel in intact cells.

## Kv1.3 Channel as a Target in Cancer Therapy. Role of Small-Molecule Membrane-Permeant Inhibitors

**Figure 4:**
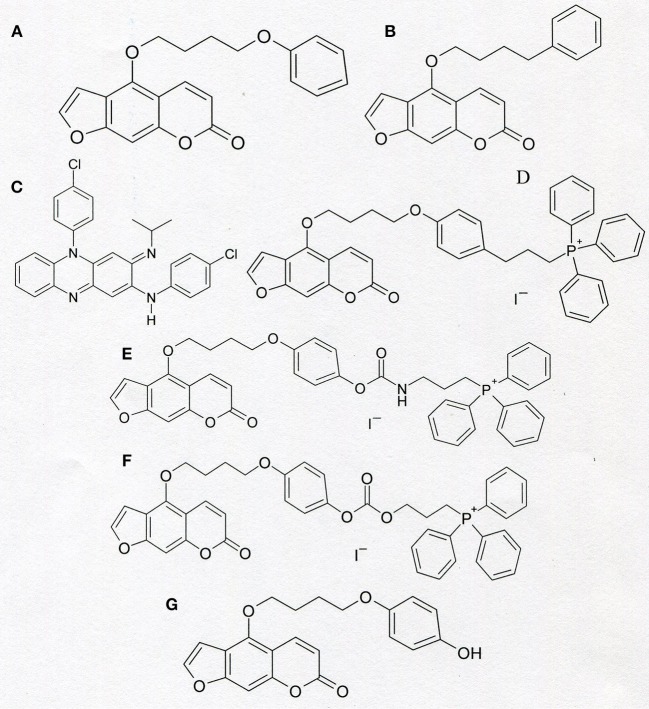
Chemical structure of PAP-1 **(A)**, Psora-4 **(B)**, clofazimine **(C)**, and of “mitochondriotropic” compounds: PAPTP **(D)**, PCARBTP **(E)**, and PCTP **(F)**. The formula of PAPOH—the product of hydrolysis of PCARBTP and PCTP is shown in the section **(G)**.

There are several specific Kv1.3 channel inhibitors that are membrane-permeant small-molecule organic compounds being able to simultaneously inhibit cancer cell proliferation (by inhibition of plasma membrane Kv1.3 channel) and to induce apoptosis of these cells (by inhibition of mito Kv1.3 channel). Two of them: 5-(4-phenylobutoxy) psoralen (Psora-4) (EC_50_ = 3 nM) and 5-(4-phenoxybutoxy) psoralen (PAP-1) (EC_50_ = 2 nM) can probably be applied as therapeutics in treatment of autoimmune diseases [(13), Figure 4]. Another promising candidate is N,5-bis(4-chlorophenyl)-3-(1-methylethylimino)-5H-phenazine-2-amine (clofazimine), which is applied in medicine since 1960's as an antibiotic in a treatment of, for example, leprosy and autoimmune disorders [(33), Figure 4]. Research performed during last 10 years provided evidence that clofazimine is also an inhibitor of Kv1.3 channel (EC_50_ = 300 nM) (33).

It was shown that an application of Psora-4, PAP-1 and clofazimine, induced apoptosis of Kv1.3 -expressing cancer cells but not of Kv1.3 channel- lacking cancer cells [(41, 42), Table 2]. The apoptosis of Kv1.3-expressing cancer cells occurred by an activation of the intracellular (mitochondrial) pathway of this process due to the inhibition of mito Kv1.3 channel in these cells (41, 42). This involved an increase of mitochondrial reactive oxygen species (ROS) production, depolarisation of the inner mitochondrial membrane, release of mitochondrial cytochrome c and cleavage of poly-ADP ribose-polymerase (PARP), followed by an activation of caspase-9 and caspase-3 (41, 42). On the other hand, membrane-impermeant peptide inhibitors, such as MgTX or ShK, were ineffective (41, 42). This would confirm that inhibition of mito Kv1.3 channel was required to induce the apoptosis of Kv1.3-expressing cancer cells (41). The ability to induce apoptosis of cancer cells was significantly augmented when the inhibitors were co-applied with inhibitors of membrane multidrug resistance transporters (MRP) (41, 42). This would prevent the inhibitors' molecules to be removed from cancer cells by MRP. Interestingly, although clofazimine is 100-times less potent Kv1.3 channel inhibitor than the other compounds mentioned above, it was the most effective inducer of apoptosis of Kv1.3-expressing cancer cells (41). This was probably due to ability to block MRP by clofazimine (41). Importantly, obtained results demonstrate that these inhibitors could induce apoptosis of Kv1.3-expressing cancer cell lines even in absence of Bax or Bak pro-apoptotic proteins, such as in case of Bax/Bak-deficient human leukemic Jurkat T cells and murine embryonic fibroblasts (MEF DKO cells) (41). Therefore, membrane-permeant Kv1.3 channel inhibitors might offer a novel option in a treatment of chemotherapeutics-resistant malignancies, by mimicking the action of pro-apoptotic proteins often down-regulated in cancer cells. However, an abundant expression of Kv1.3 channel in cancer cells is necessary to make such a treatment work. Studies performed on ten cancer cell lines of different origins: myeloid leukemia (OCIAML-3, HL-60, K562, ML-1, MOLM-13), breast adenocarcinoma (MCF-7, MDA-MB-231), colon carcinoma (DLD-1, Colo205) and neuroblastoma (SHSY5Y), showed a negative correlation between cancer cell survival upon the inhibitor application and Kv1.3 channel expression in these cells (43). In case of clofazimine such a correlation was statistically significant (43). Importantly, such a correlation was absent in case of other examined potassium channels, both Kv1.3-family related (Kv1.1) and unrelated (K_Ca_3.1 and Kv11.1) (43). Thus, the pro-apoptotic effect of clofazimine in cancer cells is really focused on inhibition of Kv1.3 channel and it requires the expression of this channel to take place (43).

**Table 2:** Anti-cancer activities of small-molecule membrane-permeant specific inhibitors of Kv1.3 channels.

**Name of the inhibitor and the value of EC_**50**_**	**Cancer cell lines affected**	**Mechanisms of anti-cancer activity**	**Maximal percentage of eliminated cancer cells**	**References**
5-(4-phenylobutoxy)- psoralen (Psora-4) EC_50_ = 3 nM	Jurkat T, human neoplastic B-CLL cells, SAOS-2, B16F10, CTLL-2-Kv1.3	Inhibition of cell proliferation combined with an induction of the mitochondrial pathway of apoptosis in cancer cells sparing normal ones.	Approximately 80% at 50 μM concentration when co-applied with inhibitors of membrane multidrug resistance transporters (MRP).	(41–43)
5-(4-phenoxybutoxy) psoralen (PAP-1) EC_50_ = 2 nM	Jurkat T, human neoplastic B-CLL cells, SAOS-2, B16F10, CTLL-2-Kv1.3	As mentioned above.	Approximately 80% at 50 μM concentration when co-applied with inhibitors of membrane multidrug resistance transporters (MRP).	(41–43)
N,5-bis (4-chloro phenyl)-3-(1-methyl ethylimino)-5H-phenazin-2-amine (clofazimine) EC_50_ = 300 nM	Jurkat T, human neoplastic B-CLL cells, SAOS-2, B16F10, As PC-1, Capan-1, Panc-1, Mia PaCa 2, Bx PC-3, Colo357, GL261, A172, LN308.	As mentioned above. Statistically significant negative correlation between cancer cells' survival and the expression of Kv1.3 channels	Approximately 90% at 50 μM concentration, reduction of tumor size of induced melanoma by 90% after 6 days of treatment, reduction of tumor weight of induced pancreatic ductal adenocarcinoma (PDAC) by more than 50% after 20 days of treatment.	(29, 41–46)
Triphenylphosphonium PAP-1 derivative -PAPTPEC_50_ = 31 nM	human neoplastic B-CLL cells, B16F10, As PC-1, Capan-1, Panc-1, Mia PaCa 2, Bx PC-3, Colo357, GL261, A172, LN308.	Induction of the mitochondrial pathway of apoptosis in cancer cells sparing normal ones.	More than 90% at 10 μM concentration, reduction of tumor size of induced melanoma by 80% after 16 days of treatment, reduction of tumor weight of induced pancreatic ductal adenocarcinoma (PDAC) by more than 50 % after 20 days of treatment.	(44–46)
Triphenylphosphonium PAP-1 derivative -PCARBTPEC_50_ = 6.5 nM for the product of hydrolysis - PAPOH	Jurkat T, human neoplastic B-CLL cells, SAOS-2, B16F10, As PC-1, Capan-1, Panc-1, Mia PaCa 2, Bx PC-3, Colo357, GL261, A172, LN308.	As mentioned above.	More than 90% at 10 μM concentration, reduction of tumor size of induced melanoma by 80% after 16 days of treatment, reduction of tumor weight of induced pancreatic ductal adenocarcinoma (PDAC) by more than 60 % after 20 days of treatment.	(44–46)
Triphenylphosphonium PAP-1 derivative -PCTPEC_50_ = 6.5 nM for the product of hydrolysis—PAPOH	Jurkat T, CTLL-2-Kv1.3, As PC-1, Capan-1, Panc-1, Bx PC-3.	As mentioned above.	More than 90% at 20 μM concentration when co-applied with inhibitors of membrane multidrug resistance transporters (MRP).	(47)

Finally, experiments performed on syngenic C57BL/6 mice showed that inhibition of Kv1.3 channel by clofazimine applied *in vivo* prevented growth of induced melanoma and reduced a tumor size by 90% after 6 days of treatment (41). Histological examination and studies on cell apoptosis showed no significant abnormalities in the function of brain, heart, lungs, small intestine, kidney, liver, and spleen tissues in clofazimine-treated mice (41). In accordance to these results, an application of the inhibitors caused a selective apoptosis of leukemic B-CLL cells without affecting T lymphocytes from patients and B and T lymphocytes from healthy donors (42). It was also shown that application of the inhibitors caused a selective apoptosis of B-CLL cells in 29 patients regardless of the stage of the disease and despite of increased expression of anti-apoptotic Bcl-2 proteins in B-CLL cells in most cases (42).

Another cancer disorder that can be targeted with clofazimine is the pancreatic ductal adenocarcinoma (PDAC) (29). Studies performed applying the real time PCR and Western blot showed up-regulated expression of Kv1.3 channel in following PDAC cell lines: AsPC-1, Capan-1, Panc-1, Mia PaCa 2, BxPC-3, and Colo357 as compared to non-tumoral human pancreatic ductal epithelial (HPDE) cells (29). An application of 10 μM clofazimine significantly reduced cell survival in case of all cancer cell lines mentioned above, but not in non-tumoral HPDE and in human umbilical vein endothelial cells (HUVEC) (29). The effect of clofazimine in AsPC-1, BxPC-3 and Colo357 cancer cells was abrogated when Kv1.3 channel was down-regulated due to transfection of the cells with Kv1.3 siRNA. These results suggest that the expression of Kv1.3 channel was necessary to induce apoptosis of PDAC cell lines (29). Membrane-impermeant inhibitors of Kv1.3 channel such as ShK and MgTX did not affect cell survival of cancer Colo357, BxPC-3, AsPC-1, and Panc-1 cells. These results indicate that mito Kv1.3 channel was the molecular target for clofazimine.

Experiments performed *in vivo* with severe combined immunodeficient (SCID) beige mice model bearing orthotopically xenotransplantated human PDAC Colo357 cells showed that after 20 days of treatment using intraperitoneally injected clofazimine at concentration of 10 nmol/g of body weight (gbw) a decrease of the tumor weight by more than 50% was observed as compared to untreated mice (29). Tissue accumulation analysis showed that clofazimine was accumulated in pancreas much more than in kidneys, spleen, lung, liver, heart, brain, and blood (29).

Recently, two derivatives of PAP-1 that can preferentially accumulate in mitochondria were synthesized. Both compounds contain a lipophilic, positively charged triphenylphosphonium (TPP^+^) group, which is linked to the psoralen backbone (44–46). The compounds' names are abbreviated as PAPTP and PCARBTP (Figure 4). Because of the positive charge within a lipophilic group, both compounds can accumulate inside mitochondria, which have a negative electric potential on the inner membrane (Δψ_m_ = −180 mV), more efficiently than uncharged molecules of PAP-1 (44). These “mitochondriotropic” properties of both compounds are responsible for their preferential inhibition of mito Kv1.3 channel (44, 45). PCARBTP applied under physiological conditions undergoes a hydrolysis to another uncharged PAP-1 derivative, PAPOH (44). PAPTP inhibits Kv1.3 channel with the EC_50_ value equal to 31 nM. PAPOH inhibits the channel with the EC_50_ value of 6.5 nM (44).

Both PAPTP and PCARBTP activate the mitochondrial pathway of apoptosis in cancer cells with expression of mito Kv1.3 channel but not in normal cells (Table 2). These compounds act more efficiently than previously applied PAP-1, clofazimine, and synthesized PAPOH (44–46).

Studies performed *in vivo* with employment of orthotropic mouse B16F10 melanoma model showed that an intraperitoneal injection of each compound caused a significant decrease of the tumor volume after 16 days since tumor cell inoculation (44). Immunohistochemical studies showed that application of PAPTP and PCARBTP did not affect healthy tissues such as brain, heart, liver, spleen, and kidney (44). Importantly, application of PAPTP and PCARBTP augmented the antitumor effect of cisplatin in murine model of melanoma (44).

Finally, the therapeutic potential of the compounds was also tested in PDAC applying a severe combined immunodeficient (SCID) beige mice model. The mice were orthotopically xenotransplantated with human PDAC Colo357 cells. The results showed that after 20 days of treatment, PAPTP and PCARBTP intraperitoneally injected at concentrations of 5 and 10 nmol/gbw, respectively, significantly reduced the tumor weight. The effect of PCARBTP on tumor weight was more pronounced than in case of clofazimine (44).

Another recently synthesized “mitochondriotropic” PAP-1 derivative with a TPP^+^ group attached to the molecule is PCTP [(47), Figure 4]. This compound is structurally related to PCARBTP (44). Similarly, to PCARBTP, PCTP also undergoes a hydrolysis that produces PAPOH (44, 47). Thus, PCARBTP and PCTP are both “prodrugs” for an actual inhibitor of mito Kv1.3 channel, which is PAPOH in both cases (44, 47). Importantly, all the compounds selectively eliminate cancer cells while sparing normal ones.

**Figure 5:**
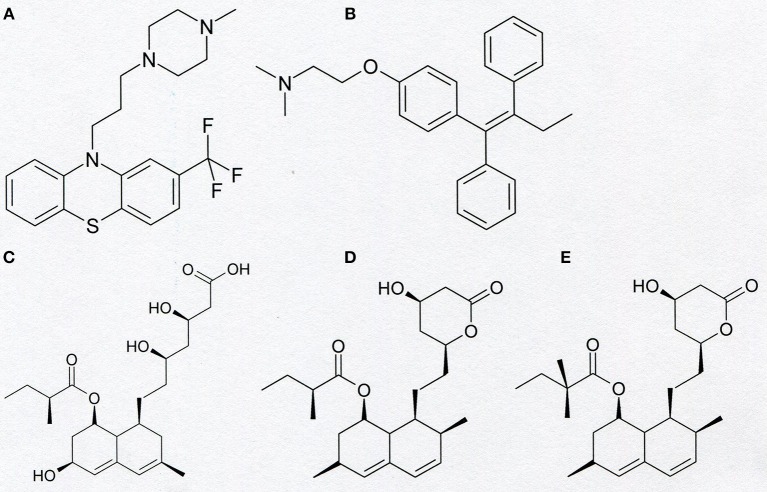
Chemical structure of trifluoperazine **(A)**, tamoxifen **(B)**, pravastatin **(C)**, lovastatin **(D)**, and simvastatin **(E)**.

Besides of clofazimine and PAP-1, there are other small-molecule organic compounds applied in medicinal therapy, which may exert anti-proliferative and pro-apoptotic effects on cancer cells due to inhibition of Kv1.3 channel. It was shown that trifluoperazine (TFP) (Figure 5), a phenothiazine derivative, which is well-known antagonist of dopamine receptors in central nervous system applied in therapy of schizophrenia, inhibits Kv1.3 channel in normal human T lymphocytes in a concentration-dependent manner (48). Almost all channels were blocked at the concentration of 50 μM (48). Interestingly, tamoxifen (TMX) (Figure 5), which is a well-known agonist of estrogenic receptors, currently used for treatment of both early and advanced estrogenic receptor-positive (ER+) breast cancer in pre- and post-menopausal women, also inhibits Kv1.3 channel in human T cells (48). It was shown that application of TMX at concentration of 20 μM caused inhibition of the channels to about 60% of the control activity (48). The inhibitory effect of TMX was similar to the effect observed upon application of TFP (48).

Other small-molecule organic compounds applied in medicinal therapy, which are inhibitors of Kv1.3 channel, are statins (Figure 5). These compounds are known as inhibitors of 3-hydroxy-3-methylglutaryl coenzyme A (HMG-CoA) reductase. Inhibition of this reductase potently inhibits the biosynthesis of cholesterol and isoprenoid metabolites. Therefore, statins are widely applied in the treatment of hypercholesterolemia and atherosclerosis (49). These compounds also exert anti-inflammatory and immunomodulatory effects that reduce the risk of cardiovascular events (49). It was shown that statins—mevastatin and simvastatin exert antiproliferative, pro-apoptotic, and reversing drug resistance effect in human colon adenocarcinoma cell line LoVo and its doxorubicin-resistant subline LoVo/Dx (49). Electrophysiological studies applying the “patch-clamp” technique showed that pravastatin, lovastatin, and simvastatin are inhibitors of Kv1.3 channel in murine thymocytes (50). A more detailed study performed on lovastatin (51) showed that this compound inhibits Kv1.3 channel expressed both in normal human T lymphocytes and in Jurkat T cells. It was shown that lovastatin inhibited Kv1.3 channel in a concentration- dependent manner (51). Moreover, it was shown that lovastatin inhibited proliferation of both normal human T lymphocytes and Jurkat T cells, in a concentration-dependent manner (51). Besides of Jurkat T cells, lovastatin inhibits proliferation and induces apoptosis of many other Kv1.3-expressing cancer cells, such as myeloid leukemia (OCIAML-3), breast adenocarcinoma (MCF-7 and MDA-MB-231), colon carcinoma (LoVo and SW-480), glioblastoma (U87), and prostate cancer (LNCaP) (52). Studies performed *in vivo* applying a mice model showed that lovastatin synergistically potentiated doxorubicin-induced cytotoxicity in colon and breast carcinoma (52). Interestingly, similarly to what was observed in the case of PAPTP and PCARBTP, lovastatin augmented antitumor effects of cisplatin in cellular and murine models of melanoma (52).

## The Inhibition of Kv1.3 Channel by Biologically Active Plant-Derived Polycyclic Compounds—a Putative Role in Anti-proliferative and Proapoptotic Activity on Cancer Cells

**Figure 6:**
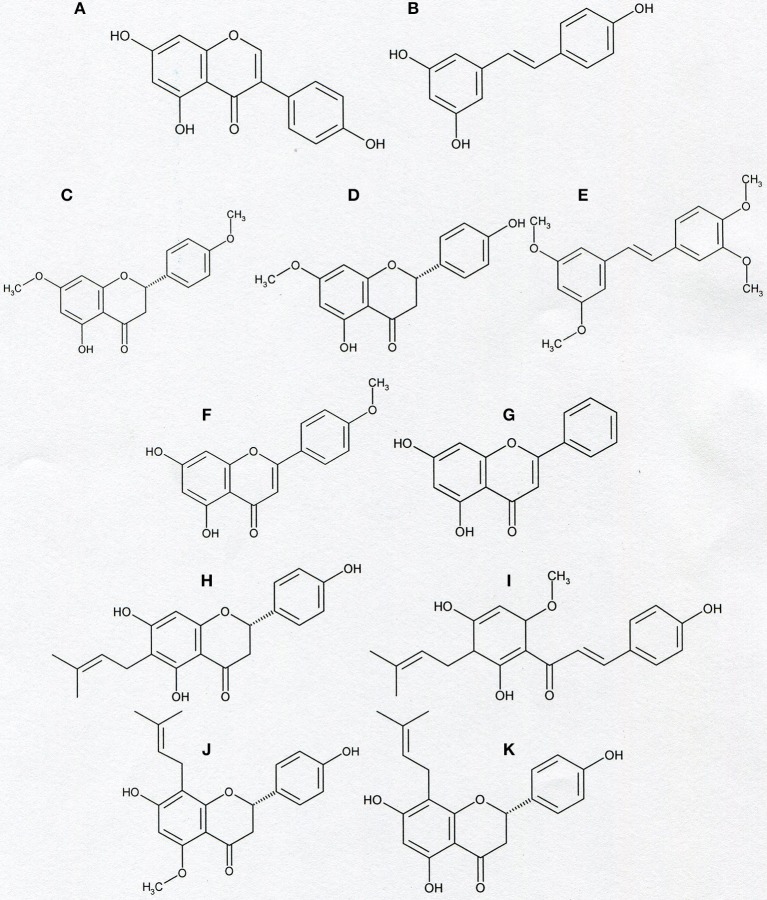
Chemical structure of genistein **(A)**, resveratrol **(B)**, naringenin-4′,7-dimethylether **(C)**, naringenin-7-methylether **(D)**, tetramethoxy- piceatannol **(E)**, acacetin **(F)**, chrysin **(G)**, and prenyl derivatives: 6-prenylnaringenin **(H)**, xanthohumol **(I)**, isoxanthohumol **(J)**, and 8-prenylnaringenin **(K)**.

There are many other Kv1.3 channel inhibitors that are membrane-permeant small-molecule organic compounds. To this group belong among others some biologically-active plant-derived polycyclic compounds from the groups of flavonoids, chalcones, and substituted stilbenes and some of their natural and synthetic derivatives (Figure 6, Table 3). These plant-derived compounds are present in everyday diet products and share anti-proliferative and pro-apoptotic effects on cancer cells. These effects are combined with a low cytotoxicity against normal cells.

**Table 3:** Inhibition of Kv1.3 channels by non-prenylated and prenylated flavonoids and substituted stilbenes.

**Name of the compound**	**Model system used for the study**	**EC_**50**_ value and magnitude of the inhibitory effect**	**References**
Genistein	Human T lymphocytes	EC_50_ = (10–40) μM Maximal inhibition−77% at 80 μM concentration	(53)
Resveratrol	Human T lymphocytes	EC_50_ = 40 μM Maximal inhibition−82% at 200 μM concentration	(54)
Naringenin-4′, 7-dimethylether	Human T lymphocytes	EC_50_ not estimated Inhibition of 96% of the currents at 30 μM Concentration	(55)
Naringenin-7-methylether	Human T lymphocytes	EC_50_ not estimated Inhibition of 71% of the currents at 30 μM concentration	(55)
Tetramethoxy- piceatannol	Human T lymphocytes	EC_50_ not estimated Inhibition of 69% of the currents at 30 μM concentration	(55)
Acacetin	Human T lymphocytes and Jurkat T cells	EC_50_ = 21 μM and 4 μM for the peak and end-of-the-pulse currents, respectively. Maximal inhibition−90% of end-of-the-pulse currents at 100 μM concentration. Inhibition of 50% of peak ramp currents at 30 μM concentration	(56, 57)
Chrysin	Jurkat T cells	Inhibition of 46% of peak ramp currents at 30 μM concentration	(57)
8-Prenyl-naringenin (8-PR)	Human T lymphocytes and Jurkat T cells	EC_50_ = 3 μM Maximal inhibition−100% at 10 μM concentration	(58)
6-Prenyl-naringenin (6-PR)	Jurkat T cells	EC_50_ = 6 μM Maximal inhibition−88% at 30 μM concentration	(57)
Xanthohumol	Jurkat T cells	EC_50_ = 3 μM Maximal inhibition−87% at 30 μM concentration	(59)
Isoxanthohumol	Jurkat T cells	EC_50_ = 8 μM Maximal inhibition−87% at 30 μM concentration	(59)

Genistein, a plant-derived isoflavone, and a substituted stilbene—resveratrol, a natural anti-cancer agent present at highest concentrations in red grapes and wine, both appeared to be inhibitors of Kv1.3 channel in human T lymphocytes. The channel inhibition occurred in a concentration-dependent manner (53, 54). It was shown in preliminary investigations that resveratrol also inhibits Kv1.3 channel in Jurkat T cells (Gasiorowska-unpublished results). The inhibition of Kv1.3 channel by genistein and resveratrol may be related to anti-proliferative and pro-apoptotic effects of these compounds on cancer cells (60, 61), however, more studies are needed to elucidate this problem in detail.

Moreover, in contrast to the precursor compounds, two synthetic methoxy- derivatives of a flavonoid naringenin (4′, 7-dimethylether and 7-methylether) and one synthetic tetramethoxy- derivative of a substituted stilbene- piceatannol also inhibit Kv1.3 channel in normal human T lymphocytes (55). Both methoxy- derivatives of naringenin also inhibit Kv1.3 channel in Jurkat T cells in a concentration-dependent manner (Gasiorowska—unpublished results). Interestingly, most of the tested compounds, like genistein, and synthetic methoxy-derivatives of naringenin and piceatannol also inhibit the activity of membrane multidrug resistance proteins MRP1 (62–64). A simultaneous inhibition of Kv1.3 channel and MRP1 proteins could facilitate pro-apoptotic effects of the compounds on Kv1.3- expressing cancer cells (55).

Another plant-derived flavonoid which inhibits Kv1.3 channel in normal human T lymphocytes and in Jurkat T cells is a methoxyflavone—acacetin (56). It was shown that acacetin inhibits Kv1.3 channel in a concentration- and time-dependent manner (56). The inhibition of Kv1.3 channel may also be involved in proapoptotic activities of acacetin (65).

Recent studies provide evidence that a natural hops-derived prenyl- derivative of naringenin−8-prenylnaringenin (8-PR) also inhibits Kv1.3 channel in Jurkat T cells in a concentration-dependent manner (58). Interestingly, 8-PR is a more potent inhibitor than non-prenylated compounds, including both synthetic methoxy—derivatives of naringenin (55, 58). The ability to inhibit Kv1.3 channel in Jurkat T cells is shared by two other hops and beer-derived prenylated compounds: a chalcone xanthohumol and a flavonoid—isoxanthohumol (59, 66, 67). In this case the channel inhibition also occurs in a concentration-dependent manner (59). The inhibitory effect of xanthohumol and isoxanthohumol was much more potent than the one exerted by non-prenylated compounds, such as genistein or resveratrol (59). These results may confirm our hypothesis that presence of prenyl group in a molecule is a factor that facilitates inhibition of Kv1.3 channel by flavonoids and chalcones (59).

The inhibition of Kv1.3 channel in Jurkat T cells by 8-PR, xanthohumol and isoxanthohumol may be involved in anti-proliferative and pro-apoptotic effects in this cancer cell line (68–72), however, more studies are needed to elucidate mechanisms of their actions.

In order to further compare the influence of prenylated and non-prenylated flavonoids on activity of Kv1.3 channel we have recently performed a study with other selected flavonoids, both prenylated and non-prenylated ones (57). The prenylated compound was another beer-derived prenylated naringenin derivative−6-prenylnaringenin (6-PR). In this study as non-prenylated flavonoids were used: acacetin, chrysin, baicalein, wogonin, and luteolin. All these compounds were tested on the same model system—Jurkat T cells at comparable concentrations using the same experimental protocol (57). Since all of the selected compounds were cytotoxic to various cancer cell lines, cytotoxic effect of these compounds against Jurkat T cells was also determined (57). Obtained results provide evidence that 6-PR inhibited Kv1.3 channel in Jurkat T cells (57). The inhibitory effect was concentration-dependent. An application of 6-PR at the highest concentration (30 μM) caused a reduction of the channel activity to ca. 12% of the control value (57). Among non-prenylated flavonoids, only acacetin and chrysin applied at 30 μM concentration inhibited the channel to about 50 and 54% of the control activity, respectively, whereas other compounds were ineffective at this concentration (57). The magnitude of the inhibitory effect exerted by acacetin and chrysin was significantly lower than in case of 6-PR application (57). These results are in accordance with our hypothesis that presence of prenyl group in a molecule facilitates inhibition of Kv1.3 channel by compounds from the groups of flavonoids and chalcones (57, 59). Results of studies on cytotoxic effect of the examined compounds showed that inhibition of Kv1.3 channel in Jurkat T cells by 6-PR, acacetin and chrysin was not related to cytotoxicity of these compounds (57).

Altogether, available results demonstrate that some biologically-active plant-derived polycyclic compounds from the groups of flavonoids, chalcones, substituted stilbenes and some of their natural and synthetic derivatives inhibit Kv1.3 channel both in normal human T lymphocytes and in Jurkat T cells. Ability to inhibit Kv1.3 channel is not a common property of these compounds, since resveratrol-related substituted stilbene, piceatannol, as well as flavonoids: naringenin, aromadendrin, baicalein, wogonin, and luteolin do not inhibit Kv1.3 channel in human normal T lymphocytes and Jurkat T cells (55, 57). The presence of prenyl group is a factor that facilitates ability of flavonoids and chalcones to inhibit Kv1.3 channel (57–59). However, a contribution of inhibition of Kv1.3 channels to the total anti-proliferative and pro-apoptotic effects of these compounds (68–74) needs further studies to be determined.

## How can Small-Molecule Organic Inhibitors of Kv1.3 Channel Selectively Kill cancer Cells Without Affecting Normal Ones?

**Figure 7:**
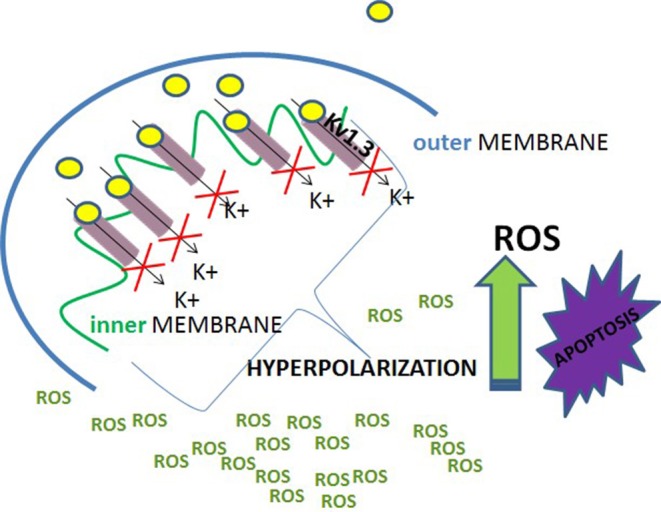
A scheme of selective activation of the mitochondrial pathway of apoptosis of Kv1.3 channels' expressing cancer cells.

The answer to that interesting question is very important. It is likely that killing selectivity is a result of up-regulation of Kv1.3 channel in these cells, which is observed, for example, in B-CLL cells (20). In case of PDAC cell lines an inverse correlation between the EC_50_ values for the induction of apoptosis by PAPTP and PCARBTP and expression of Kv1.3 channel was obtained (44). This is in agreement with a negative correlation between survival of cancer cells upon application of clofazimine and expression of Kv1.3 channel in these cells (43). However, it must be pointed out that in contrast to what was observed for Jurkat T cells, normal human T lymphocytes, even effector memory T cells (T_EM_), which predominantly up-regulate Kv1.3 channel upon activation, did not undergo the apoptosis upon an application of the compounds (44). Therefore, selective apoptosis of cancer cells cannot be solely due to the up-regulation of Kv1.3 channel. Interestingly, the pro-apoptotic effect of the compounds on cancer cells was abolished upon application of anti-oxidants, such as, for example, N-acetyl cysteine (NAC) (44). This indicates that the production of ROS is also involved in a mechanism of the pro-apoptotic activity of the compounds. It is well-known that ROS are produced by mitochondria both in normal and in cancer cells. However, their basal concentration in mitochondria of normal cells is low. Superoxide anion-radicals (${\text{O}}_{2}^{-}$) are either oxidized to molecular oxygen (O_2_) or reduced to hydrogen peroxide (H_2_O_2_) by the enzyme superoxide dismutase (SOD2) (75). H_2_O_2_ is then reduced to H_2_O by the enzymes—glutathione peroxidases (GPX) and peroxiredoxins, which use reduced glutathione (GSH) as a ROS- reducing agent. The ROS- oxidized glutathione (GSSG) is then reduced to GSH by the enzyme glutathione reductase (75). On the other hand, since ROS are needed for cancer development and metastasis, their basal level in cancer cell mitochondria is significantly higher than in normal cells. This is due to increased ROS production combined with a reduced ability for ROS degradation (75). Elevated ROS release to the cytosol promotes, in turn, cancer development, and metastasis by an activation of the hypoxia-inducible factor 1α (HIF1α) (75). On the other hand, it is known that inhibition of mito Kv1.3 channel causes a transient hyperpolarisation of the inner mitochondrial membrane and this hyperpolarisation facilitates the production of ROS by mitochondria (37). Taking into account that cancer cells, such as B-CLL cells, up-regulate Kv1.3 channel, including mito Kv1.3 channel, one can assume that this mito Kv1.3 channel inhibition- induced hyperpolarisation is more pronounced in cancer cells than in normal ones. Thus, one can assume that the mito Kv1.3 channel inhibition- induced ROS production (mito Kv1.3—ROS) is also elevated in cancer cells. The elevated mito Kv1.3—ROS combined with a high basal ROS level in cancer cell mitochondria leads to an excessive “oxidative stress,” which may activate the mitochondrial pathway of apoptosis of cancer cells (75) (Figure 7).

On the other hand, one can assume that the mito Kv1.3—ROS in normal cells is lower than in cancer cells. The reduced mito Kv1.3—ROS combined with a low basal ROS level in normal cell mitochondria enables a normal cell to avoid the excessive “oxidative stress” leading to the apoptosis (44). Importantly, neither elevated mito Kv1.3—ROS nor high basal ROS level can induce cancer cell apoptosis when acting alone. Therefore, down-regulation of Kv1.3 channel, which reduces the mito Kv1.3—ROS, protects cancer cells from the apoptosis, whereas restoration of the channel expression is enough to re-sensitize these cell to the apoptosis. Alternatively, an application of antioxidants or membrane-permeant ROS scavengers: superoxide dismutase (PEG—SOD) and catalase (PEG—CAT), which reduces the basal ROS level in cancer cells, abolishes the pro-apoptotic effect of inhibition of mito Kv1.3 channel by the compounds (42). On the other hand, a low basal ROS level in normal human T lymphocytes protects these cells from apoptosis even in the case of up-regulation of Kv1.3 channel in activated effector memory T cells (T_EM_) (44).

In summary, potent, small-molecule membrane-permeant inhibitors of Kv1.3 channel can selectively induce apoptosis of cancer cells by mito Kv1.3 channel inhibition, which activates the mitochondrial pathway of this process. This is due to a combination of elevated mito Kv1.3 channel inhibition- induced ROS production and high basal ROS level in cancer cell mitochondria. Such a situation does not occur in case of normal cells, even in those, which predominantly up-regulate Kv1.3 channel. Therefore, normal cells do not undergo apoptosis upon application of the inhibitors.

## Practical Precautions

However, one should be cautious when considering a possible application of small-molecule organic inhibitors of Kv1.3 channel in a clinical practice. The first problem is down-regulation of Kv1.3 channel in cancer cells, which leads to apoptosis resistance. This requires an additional pre-treatment to restore the channel expression and re-sensitize cancer cells to apoptotic signals before the treatment with inhibitors of the channel. It is known that expression of the channel can be restored in cancer cells upon application of the gene demethylation agent (30). However, it remains to be elucidated whether such a restoration leads to a re-sensitization of these cells to apoptotic stimuli. Another problem is the question of applied dose. An application of PAPTP and PCARBTP induced the apoptosis of PDAC cell line PANC-1 with the EC_50_ values equal to 3.0 and 6.3 μM, respectively (45). On the other hand, an application of each compound at a sub-lethal concentration of 100 nM, which is high enough to inhibit mito Kv1.3 channel, exerted an unexpected stimulatory effect on the proliferation of cancer cells (45). It was shown that the relative number of cells in the S phase, which is a parameter widely used to describe the proliferative capacity of cells, was significantly increased upon an application of PAPTP and PCARBTP (45). On the other hand, an application of a membrane-impermeant peptide inhibitor of plasma membrane Kv1.3 channel—ShK—inhibited proliferation of cancer cells, such as it was expected (45). Moreover, an application of clofazimine at a sub-lethal concentration of 1 μM, which is high enough to inhibit both plasma-membrane and mito Kv1.3 channels, does not change the relative number of cells in the S phase (45). These results suggest that the unexpected pro-proliferative effect of PAPTP and PCARBTP is due to a preferred inhibition of mito Kv1.3 channel. It is well-known that inhibition of mito Kv1.3 channel causes a transient hyperpolarisation of the inner mitochondrial membrane and the hyperpolarisation- induced production of ROS by mitochondria (37). In accordance with this phenomenon, an application of PAPTP and PCARBTP at a concentration of 100 nM stimulated the ROS production by mitochondria of PANC-1 cells (45). This effect was abolished when the cells were pre-incubated with ROS scavengers, such as anti-oxidant N-acetyl cysteine (NAC) or mitochondriotropic Mitotempo (45). Interestingly, pre-incubation with NAC or Mitotempo also abolished the pro-proliferative effect of the compounds (45). This indicates that this pro-proliferative effect is due to the mito-Kv1.3 channel inhibition-induced ROS production (mito Kv1.3—ROS) in PDAC cancer cells. The same mito Kv1.3—ROS phenomenon is responsible for the induction of apoptosis of cancer cells by the same compounds when applied at micromolar concentrations (41, 42, 44, 45). A similar process was also observed in case of other PDAC cell line Colo357 treated with PAPTP and PCARBTP applied at concentrations of 100 and 35 nM, respectively (45). In summary, these results demonstrate that application of the compounds may exert either a pro-proliferative effect (at sub-micromolar concentrations) or a pro-apoptotic effect (at low micromolar concentrations) on cancer cells. In order to assure the prevalence of the desired pro-apoptotic effect, the concentration equal to at least 10 μM is necessary.

The ability of above-mentioned compounds to induce cancer cell apoptosis is not only concentration- but also tissue-dependent. Pharmacokinetic studies provide evidence that PAPTP, PAP-OH (the product of hydrolysis of PCARBTP), and PAP-1 are differently distributed among tissues—they were found in liver, spleen, kidney, blood, heart, and tumor tissues, but they were completely absent in brain (44). This is probably due to inability of these compounds to cross the blood-brain-barrier (44). In accordance to this observation, injections of PAPTP, PCARBTP and clofazimine did not reduce the growth of a brain glioma in the group of glioma-injected mice (46). This inefficiency was not due to absence of Kv1.3 channel. Immunohistochemical studies revealed presence of Kv1.3 channel, both in the plasma membrane and in mitochondria, in murine GL261 and in human A172 and LN308 glioma cell lines (46). Moreover, it was shown that application of PAPTP, PCARBTP and clofazimine at concentrations from 5 to 10 μM caused death of almost all cells from these lines due to activation of the mitochondrial pathway of apoptosis (46). These data indicate that the compounds induce apoptosis of Kv1.3 channel- expressing glioma cell lines as potently as in case of other cancer cell lines expressing Kv1.3 channel (29, 42–47). Nevertheless, since the compounds are unable to cross the blood-brain barrier and reach their molecular targets in the brain, they are ineffective when applied *in vivo*.

## Conclusion Remarks

Results presented in this review demonstrate that Kv1.3 channel may be useful molecular target in cancer therapy. In order to exert an anti-proliferative and pro-apoptotic effect on cancer cells it is necessary to inhibit the channel expressed both in the plasma membrane and in mitochondria. Therefore, a high lipophilicity of the inhibitors is required. Furthermore, since the anti-cancer therapy may fail due to a multidrug resistance, ability of the compounds to simultaneously inhibit membrane multidrug resistance proteins is also recommended. Finally, ability to cross the blood-brain-barrier is necessary for application of the compounds in therapy of brain tumors. At the present, there are no inhibitors of Kv1.3 channel, which meet all the criteria listed above. On the other hand, it must be pointed out that the channel inhibition offers a possibility to selectively eliminate Kv1.3 channel- expressing cancer cells while sparing normal cells and healthy organs. This advantage may support all the effort to discover new potent inhibitors of Kv1.3 channel, which could be effectively applied in anti-cancer therapy.

## Author Contributions

AT, AP-L, and KS-P wrote the manuscript and drew the figures. KM critically revised and corrected the manuscript.

### Conflict of Interest

The authors declare that the research was conducted in the absence of any commercial or financial relationships that could be construed as a potential conflict of interest.
